# Supplementary material for: Isolation, Antibacterial Activity and Molecular Identification of Avocado Rhizosphere Actinobacteria as Potential Biocontrol Agents of Xanthomonas sp
Source: Microorganisms. 2024 Oct 31;12(11):2199. doi: 10.3390/microorganisms12112199 (PMC11596542; doi:10.3390/microorganisms12112199)
Supplement: Supplementary file 1 [file microorganisms-12-02199-s001.zip › microorganisms-3275844-supplementary.pdf]

Supplementary material

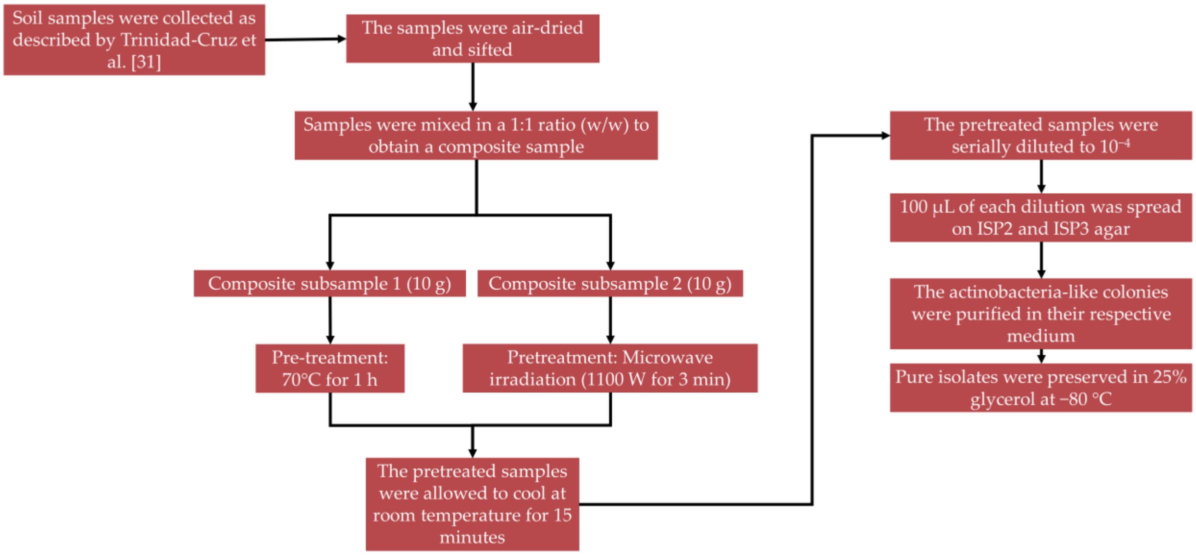

Figure S1. Flowchart of the actinobacteria isolation process from a composite soil sample obtained from the rhizosphere of avocado trees cv. Hass (Trinidad-Cruz et al. [31]).

31 Trinidad-Cruz, J.R.; Rincón-Enríquez, G.; Evangelista-Martínez, Z.; Guízar-González, C.; Enríquez-Vara, J.N.; López-Pérez, L.; Quiñones-Aguilar, E.E. Actinobacteria from avocado rhizosphere: antagonistic activity against *Colletotrichum gloeosporioides* and *Xanthomonas* sp. *Terra Latinoam.* **2021**, *39*, e802. https://doi.org/10.28940/terra.v39i0.802.
